# Supplementary material for: Structural mechanism of R2D2 and Loqs-PD synergistic modulation on DmDcr-2 oligomers
Source: Nat Commun. 2023 Aug 26;14:5228. doi: 10.1038/s41467-023-40919-1 (PMC10460399; doi:10.1038/s41467-023-40919-1)
Supplement: Supplementary file 1 — Supplementary Information [file 41467_2023_40919_MOESM1_ESM.pdf]

Structural mechanism of R2D2 and Loqs-PD synergistic modulation on *Dm*Dcr-2 oligomers

Supplementary Figures

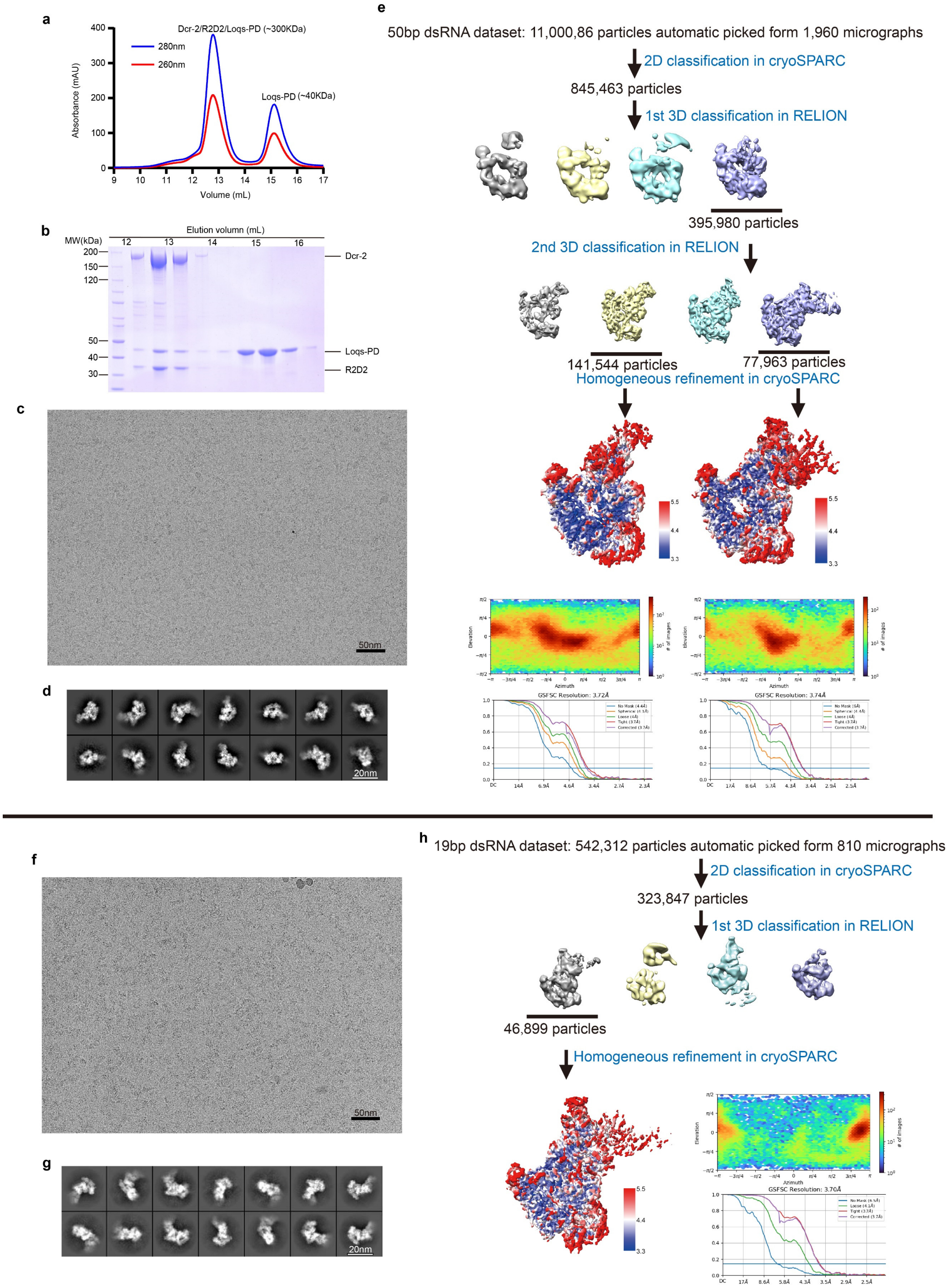

Supplementary Figure 1. **Protein purification and Cryo-EM image processing workflow of *Dm*Dcr-2^{DDNN}/R2D2/Loqs-PD complex with 50bp and 19bp dsRNA.**

a, Size exclusion chromatography results of *Dm*Dcr-2/R2D2/Loqs-PD complex. **b**, SDS-PAGE of the protein fractions from **a**. Source data are provided as a Source Data file. **c**, A representative cryo-EM image of the 50bp-complex. **d**, Representative views of 2D class averages of the 50bp-complex. **e**, Flowchart of cryo-EM data processing of the 50bp-complex. **f**, A representative cryo-EM image of the 19bp-complex dataset. **g**, Representative views of 2D class averages of the 19bp-complex. **h**, Flowchart of cryo-EM data processing of the 19bp-complex dataset.

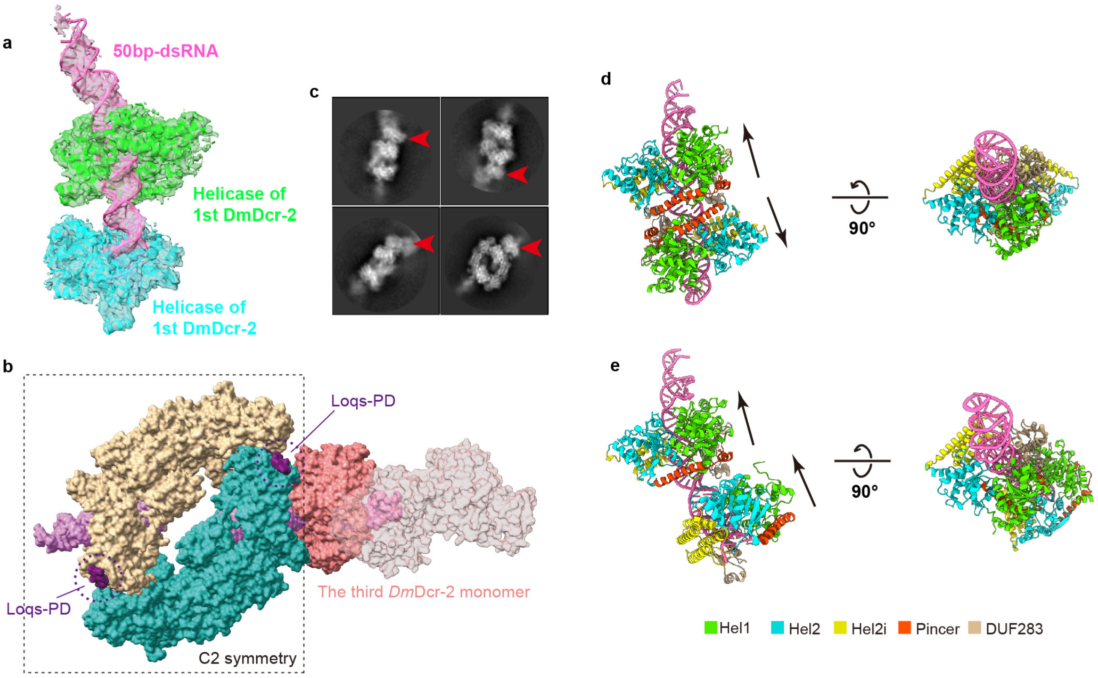

Supplementary Figure 2. **R2D2 can affect the arrangement mode of the Helicase domain on dsRNA. a**. Cryo-EM density and fitted models show that two Helicase domains can bind to the same dsRNA. **b-c**. The 3D model and selected 2D averages from the previously published dataset of *Dm*Dcr-2/Loqs-PD/50bp-dsRNA/ATP (red arrows indicate the density of the third *Dm*Dcr-2). **d**. The Helicase domains can be arranged in a tail-to-tail mode on the same dsRNA, as seen in PDB-7W0D. **e**. The addition of R2D2 to the original components results in the Helicase domain adopting a head-to-tail mode in the Dimer state. Therefore, the Helicase domain can be arranged in both head-to-tail and tail-to-tail modes.

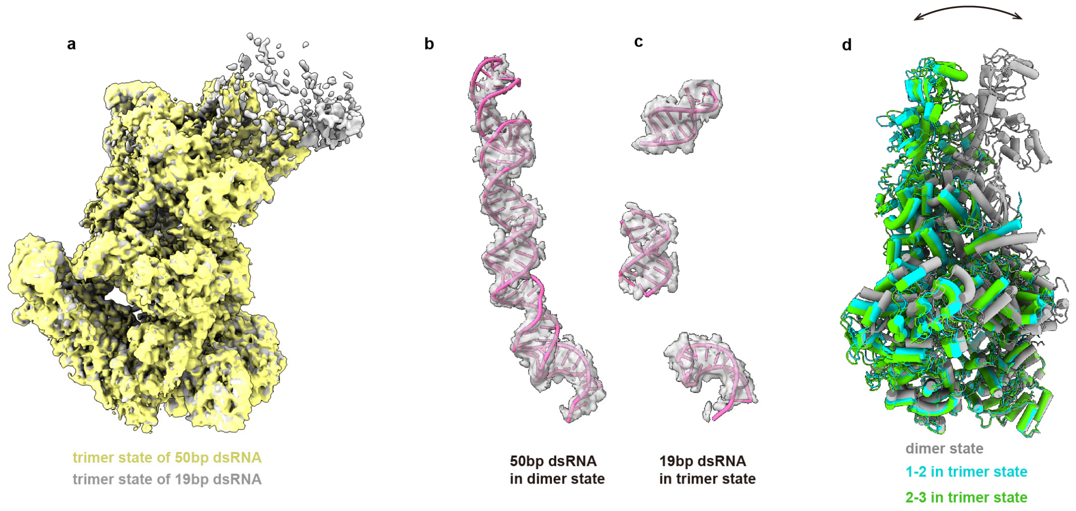

Supplementary Figure 3. **Differences of dsRNA density and protein state in the oligomers.**
a, Superposition of two Trimer state structures from 50bp and 19bp dsRNA. The two structures are identical. **b**, The dsRNA density and fitted model of 50bp dsRNA in dimer state. The protein density was erased from the cryo-EM maps. **c**, The dsRNA density and fitted model of 19bp dsRNA in trimer state. **d**, The Trimer state model was separated into two dimers and then superimposed together by aligning their first *Dm*Dcr-2. grey: Dimer state; cyan: the first two *Dm*Dcr-2 monomers in Trimer state; lime: the last two *Dm*Dcr-2 monomers in Trimer state.

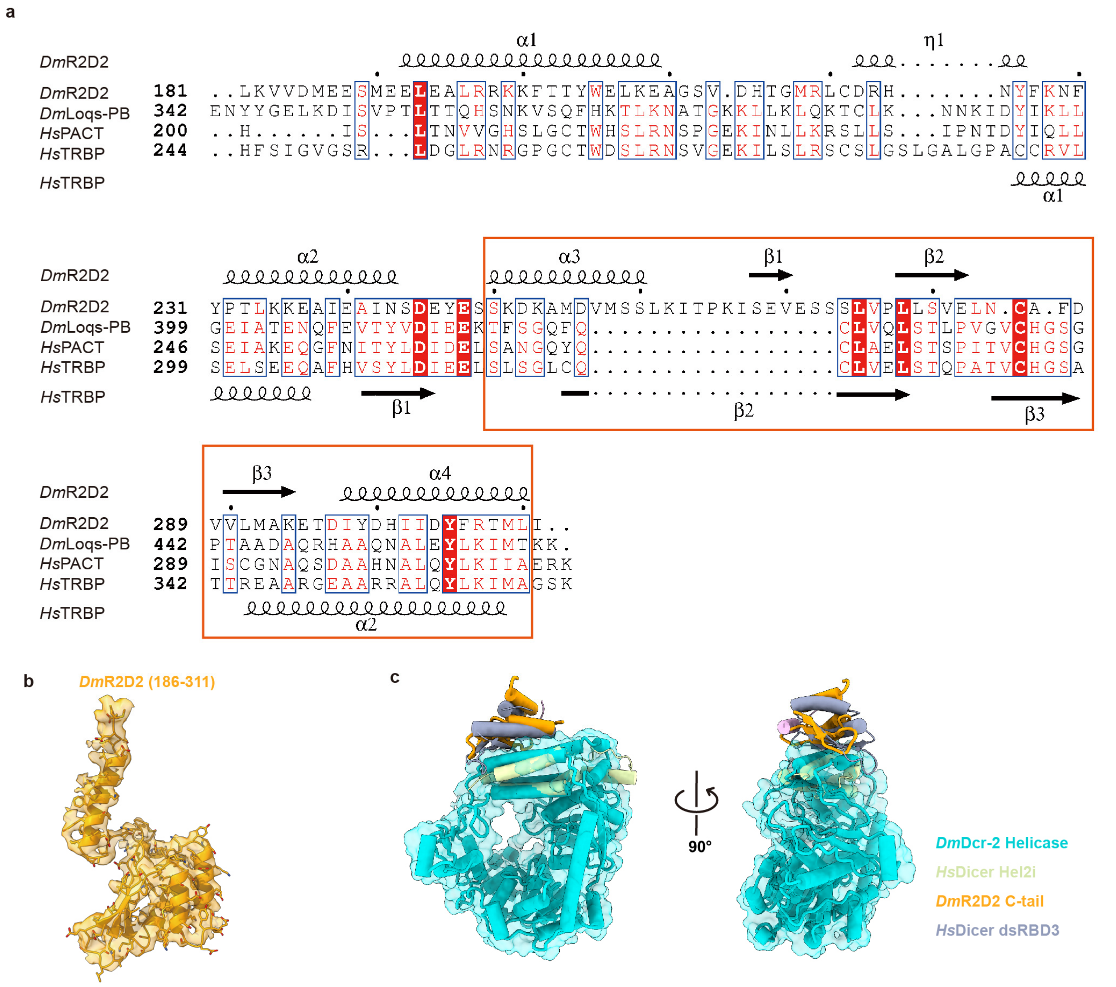

Supplementary Figure 4. Sequence and structure fractures C terminus of R2D2. a, The sequence alignment of the C-terminus of R2D2 with other Dicer cofactors containing type B dsRBDs is shown. The top panel displays the secondary structure of R2D2, and the bottom panel shows the secondary structure of TRBP based on PDB-4WYQ. The region highlighted by the orange box indicates the third dsRBD of R2D2, while the yellow box indicates the mutated region of the "bridge-helix". **b**, The cryo-EM density of aa 186-311 of R2D2 is shown, along with the fitted atomic model. **c**, The binding site of R2D2 is superimposed with that of TRBP (PDB-4WYQ), aligned by the Hel2i domain. The *Dm*Dcr-2 helicase is depicted with a surface at 50% transparency. The third dsRBD of R2D2 and TRBP exhibit similar structures and binding sites on Dicers.

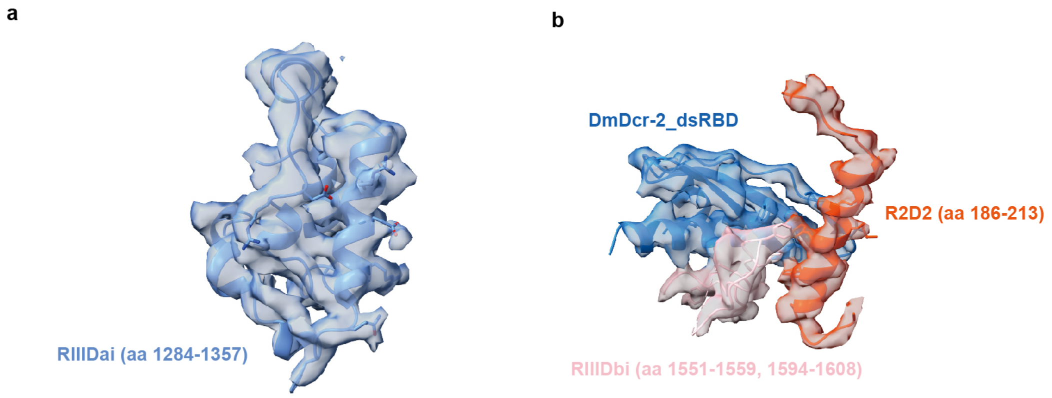

Supplementary Figure 5. **The cryo-EM density of RIIIDai and RIIIDbi of Dcr-2 with the fitted models. a**, The cryo-EM density of RIIIDai with the fitted model. **b**, The cryo-EM density near RIIIDbi with the fitted model.

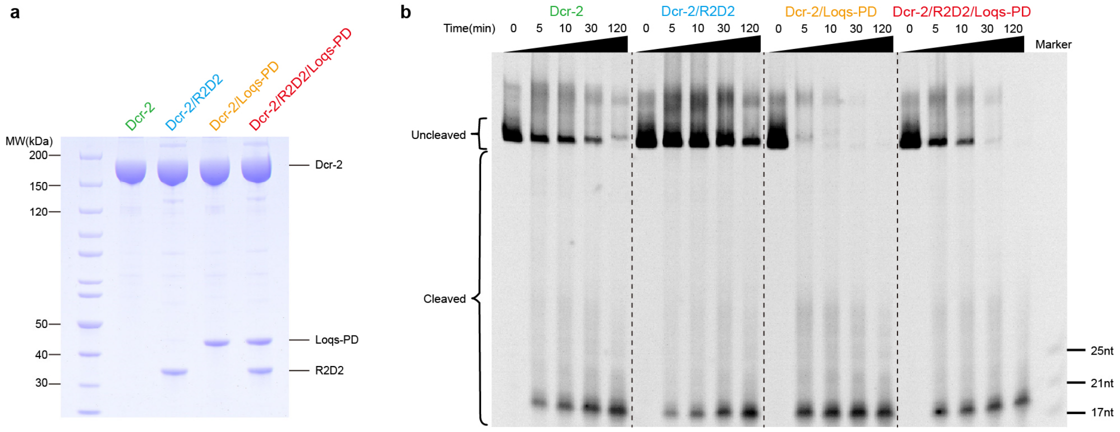

Supplementary Figure 6. **Cleavage assays of *Dm*Dcr-2, *Dm*Dcr-2/Loqs-PD, *Dm*Dcr-2/R2D2, *Dm*Dcr-2/R2D2/Loqs-PD complexes. a**, SDS-PAGE of *Dm*Dcr-2, *Dm*Dcr-2/Loqs-PD, *Dm*Dcr-2/R2D2, *Dm*Dcr-2/R2D2/Loqs-PD using in the cleavage assays, 2 times repeated independently with similar result. **b**, Cleavage assays of *Dm*Dcr-2, *Dm*Dcr-2/Loqs-PD, *Dm*Dcr-2/R2D2, *Dm*Dcr-2/R2D2/Loqs-PD with 106-bp blunt (BLT) termini dsRNA(5'-GGCAAUGAAAGACGGUGAG CUGGUGAUAUGGGAUAGUGUUCACCCUUGUUACACCGUUUUCCAUGAGCAAACUG AAACGUUUUCAUCGCUCUGGAGUGAAUACCAC-3' and 5'-GUGGUAUUCACUCCA GAGCGAUGAAAACGUUUCAGUUUGCUCAUGGAAAACGGUGUAACAAGGGUGAACA CUAUCCCAUAUCACCAGCUCACCGUCUUUCAUUGCC-3') with 5 mM ATP and 5 mM $MgCl_2$ in the cleavage assay buffer (100 mM Tris-8.0, 100 mM NaCl, 1 mM DTT) , 25 °C for up to 120 min. Products were resolved on a 12% polyacrylamide denaturing gel, 4 times repeated independently with similar result. The Neb microRNA Marker A (N2102) is in the leftmost lane. Source data are provided as a Source Data file.

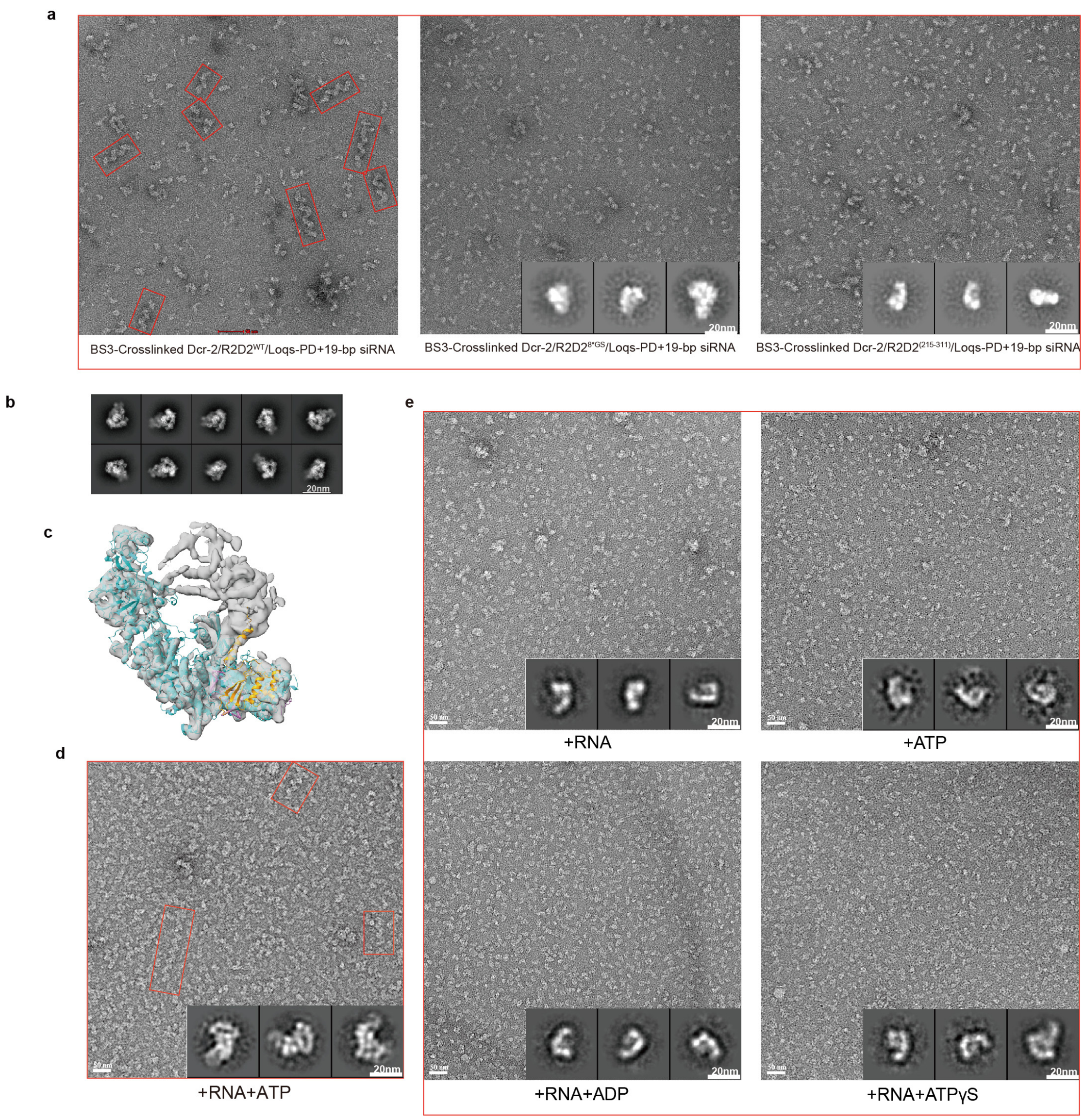

Supplementary Figure 7. **Factors that play important roles in the oligomerization of the Dcr-2/R2D2/Loqs-PD+19-bp siRNA complex.** **a**, Negative-staining micrographs of the BS3-crosslinked Dcr-2/R2D2/Loqs-PD+19-bp siRNA complexes with different constructs of R2D2. 2D averages are at the lower right corner, 2 times repeated independently with similar result. **b-c**, The selected 2D averages and 3D density map of the oligomer with the "bridge-helix" mutation are shown. With the exception of the first *Dm*Dcr-2, the densities of the other parts are unclear. Due to the overall structure's instability, more data (here, 2,346 cryo-EM micrographs with the same parameters as the 19bp-complex dataset) yield a lower resolution density map. **d**, A typical negative-staining micrograph showing oligomers (19-bp dsRNA used here), with three fibers marked in red boxes. The 2D averages shows that almost all particles are oligomers, 3 times repeated independently with similar result. **e**, *Dm*Dcr-2/R2D2/Loqs-PD complexes were incubated with various components (RNA only, ATP only, RNA and ADP, RNA and ATPγS, the RNA using the 19-bp dsRNA) in a buffer containing 150 mM NaCl, 20 mM Tris-HCl pH 8.0, and 5 mM MgCl2 for 2 hours before negative-staining, 3 times repeated independently with similar result. The 2D class-averages obtained showed that none of the above components alone or in combination will form large oligomers.

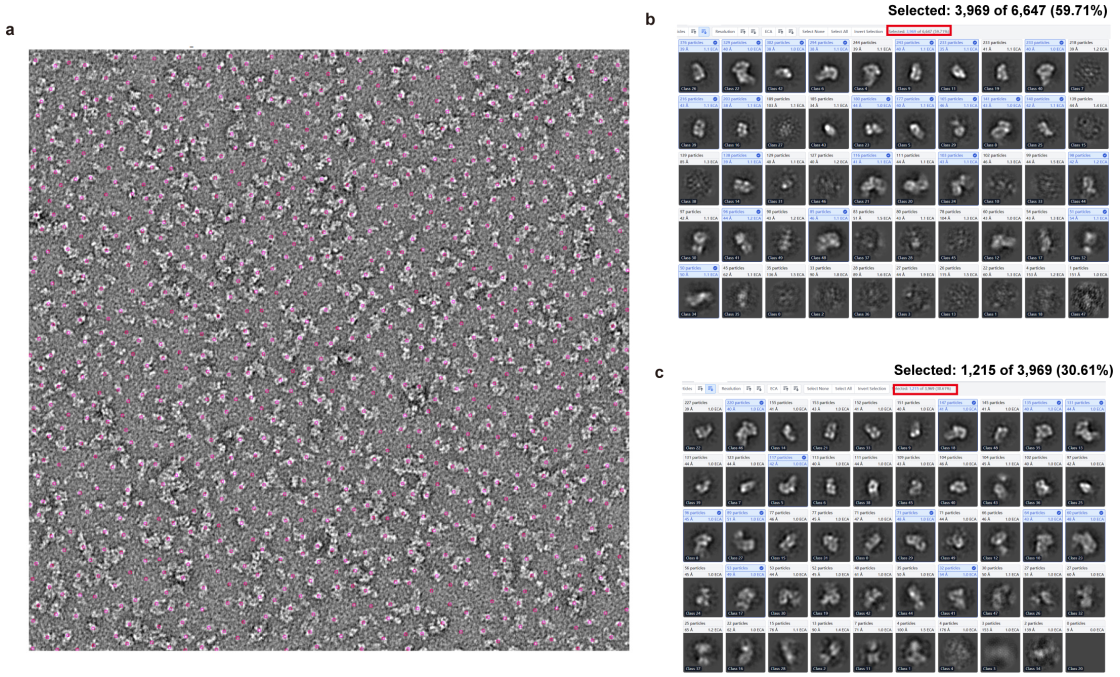

Supplementary Figure 8. One approach for determining the ratio of oligomers is through negative-staining electron microscopy. The process involves the following steps: **a**, acquiring raw micrographs and picking particles, **b**, performing the first round of 2D class-averages to remove any contaminants, and **c**, calculating the ratio of oligomers based on the resulting 2D averages (30.61% here).

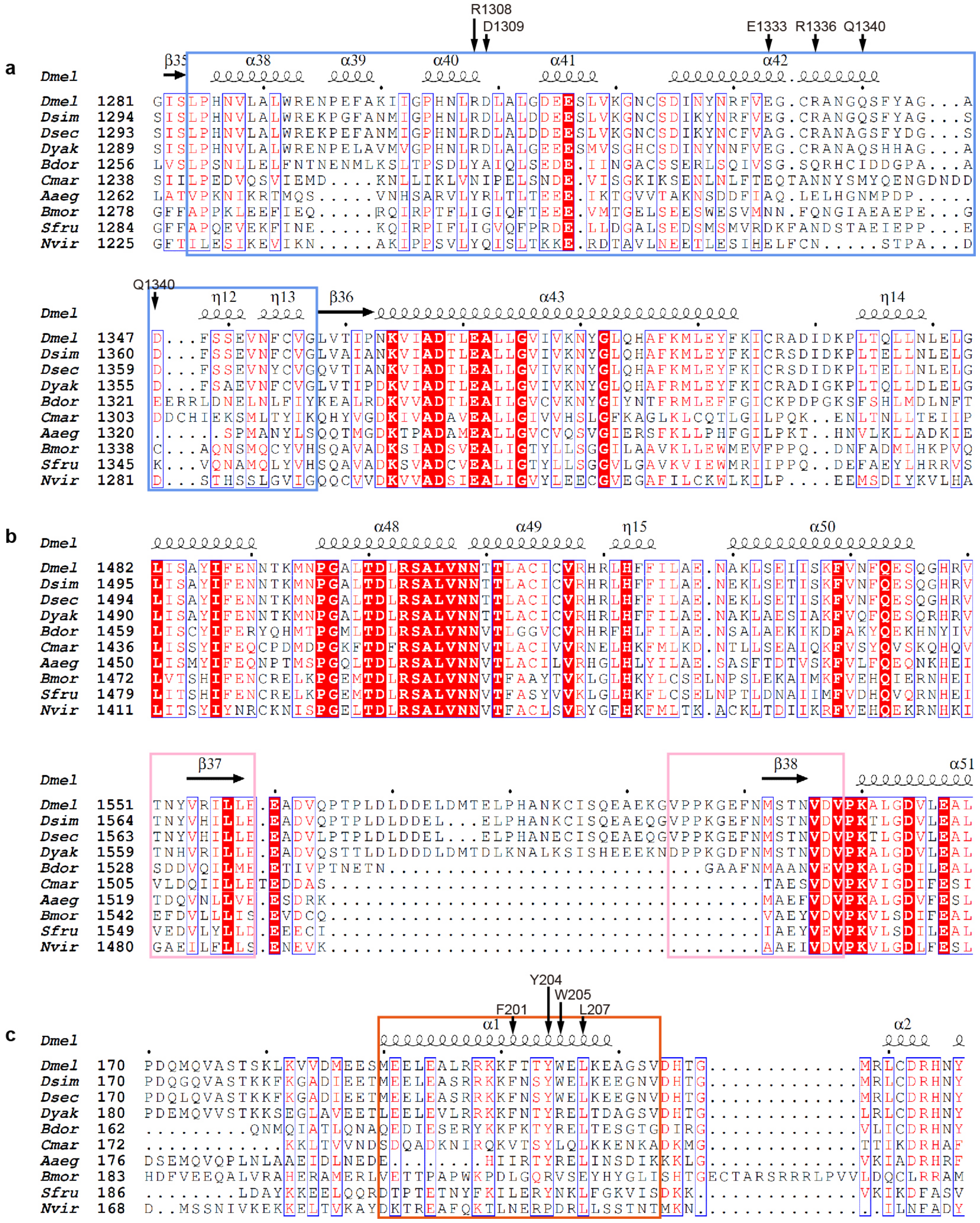

Supplementary Figure 9. **RIIIDai and RIIIDbi of Dcr-2, bridge helix of R2D2 show high conservation in *Drosophila*. a-c**, Sequence alignment of the RIIIDai, RIIIDbi, and R2D2 (blue, pink and orange boxes respectively representing their modeled structures in **Supplementary Figure 5**). The labeled amino acids correspond to **Fig. 3d** and **3f**. The alignment result shows that these regions are relatively conserved in *Drosophila; Dmel, Drosophila melanogaster; Dsim, Drosophila simulans; Dsec, Drosophila sechellia; Dyak, Drosophila yakuba; Bdor, Bactrocera dorsalis; Cmar, Clunio marinus; Aaeg, Aedes aegypti; Bmor, Bombyx mori; Sfru, Spodoptera frugiperda; Nvir, Nezara viridula*.

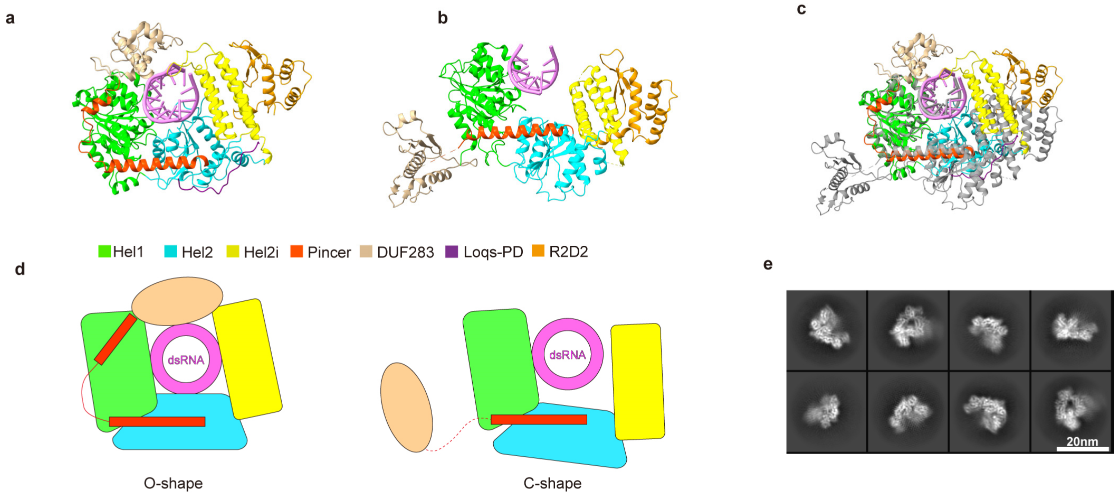

Supplementary Figure 10. Loqs-PD is likely to facilitate oligomer formation. a-b, Overview of the Helicase domain in the 1st monomer of the 19bp-dsRNA trimer state (**a**) and the *Dm*Dcr-2/Loqs-PD/siRNA complex (PDB:7V6C) (**b**). The dsRNA duplex alone cannot induce the conformational changes of the Helicase-DUF283 domains in *Dm*Dcr-2/R2D2 complex that are necessary for the oligomeric process. Therefore, Loqs-PD is crucial in initiating the oligomerization process. **c**, Superposition of Helicase domain in **a** and **b** by aligned Hel1, with the Helicase from **a** colored by domains and the Helicase from **b** colored in gray. **d**, Model of Helicase domain in **a** and **b** as O-shape and C-shape. **e**, 2D class-averages of *Dm*Dcr-2/R2D2/siRNA-duplex samples incubated with ATP and $MgCl_2$ from 1,585 cryo-EM micrographs. The components and experimental steps were identical to the oligomer formation experiment, except for the absence of Loqs-PD. When Loqs-PD is absent, the majority of complexes fail to oligomerize.

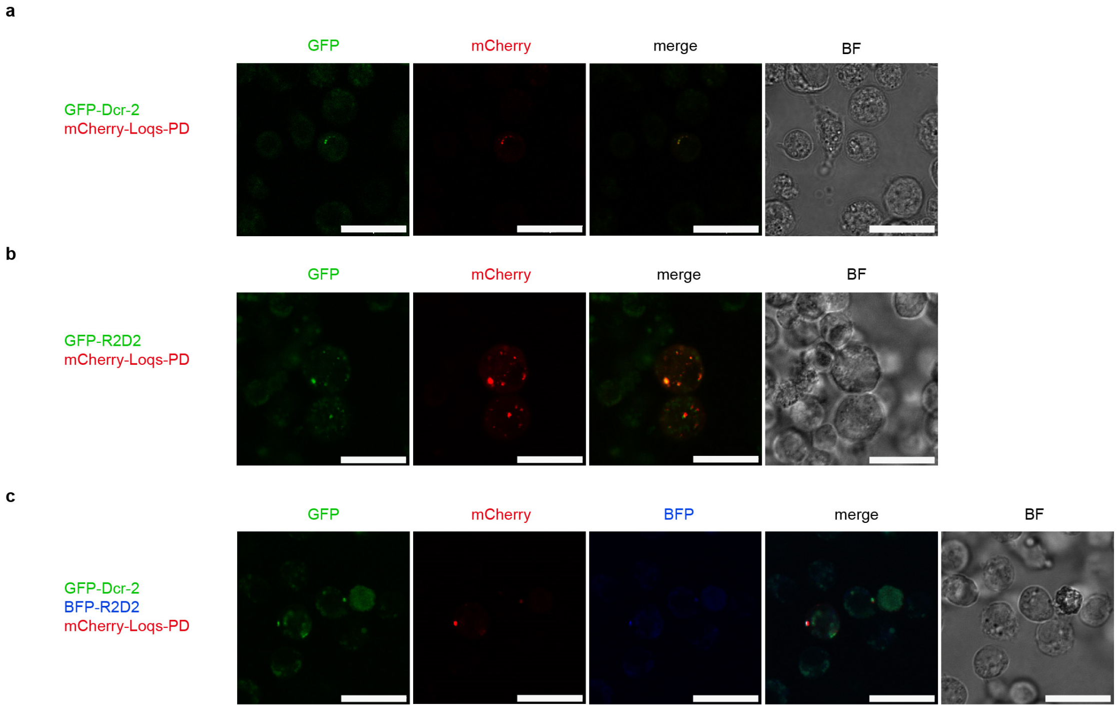

Supplementary Figure 11. The co-localization of *Dm*Dcr-2, R2D2, and Loqs-PD *in vivo*. a, Fluorescence images of co-transfected GFP-Dcr-2 and mCherry-Loqs-PD in S2 cell. Scale bars, 20µm. **b**, Fluorescence images of co-transfected GFP-R2D2 and mCherry-Loqs-PD in S2 cell. Scale bars, 20µm. **c**, Fluorescence images of co-transfected GFP-Dcr-2, BFP-R2D2, and mCherry-Loqs-PD in S2 cell. Scale bars, 20µm. The fluorescence results were repeated independently 3 times independently with similar result.

Supplementary Table 1. Cryo-EM data collection, processing, model refinement and validation statistics.

	50bp-Dimer	50bp-Trimer	19bp-Trimer
PDB ID	8HF0	-	8HF1
EMDB ID	EMD-34707	EMD-34709	EMD-34708
**Data collection and processing**			
Microscope		Titan Krios	
Detector		Gatan K3 with GIF Quantum (20eV slit)	
CS (mm)	2.7	2.7	0.01
Magnification	81K	81K	64K
Pixel size (Å)	1.0742	1.0742	1.08
Electron dost (e^{-}/ $Å^{2}$)	50(32 frames)	50(32 frames)	50(32 frames)
Defocus range (μm)	-1.5 ~ -2.0	-1.5 ~ -2.0	-1.5 ~ -2.0
Micrograph Number	1,960	1,960	810
**Reconstruction**			
Software		RELION-3.1, cryoSPARC-3.2	
Particles picked	1,100,086	1,100,086	542,312
Particles refinement	141,544	77,693	46,899
Symmetry	C1	C1	C1
Resolution (Å)	3.72	3.74	3.70
Sharpening B-factor ($Å^{2}$)	127.0	98.1	88.8
**Refinement**			
Software		PHENIX-1.19.2368	
Model composition			
Number of atoms	29,510		42,379
Protein residues	3,388		5,075
Nucleotides	97		60
B factors (Protein/Nucleotide/Ligand)	122.41/184.77/0		218.42/203.12/0
Bonds RMSD			
Bonds lengths (Å)	0.003		0.007
Bonds angles (°)	0.573		0.748
**Validation**			
MolProbity score	1.75		1.99
Clash score	10.27		14.00
Rotamer outliers (%)	0.00		0.00
C-beta outliers (%)	0.03		0.00
Ramachandran plot			
Favored (%)	96.56		95.21
Allowed (%)	3.41		4.77
Outlier (%)	0.03		0.02
Model vs. Data			
CC mask/box	0.79/0.88		0.79/0.80
