# Supplementary material for: Structural mechanism of R2D2 and Loqs-PD synergistic modulation on DmDcr-2 oligomers
Source: Nat Commun. 2023 Aug 26;14:5228. doi: 10.1038/s41467-023-40919-1 (PMC10460399; doi:10.1038/s41467-023-40919-1)
Supplement: Supplementary file 3 — Reporting Summary [file 41467_2023_40919_MOESM3_ESM.pdf]

Corresponding author(s): Jia Wang�Jinbiao Ma

Last updated by author(s): Jun 4, 2023

Reporting Summary

Nature Portfolio wishes to improve the reproducibility of the work that we publish. This form provides structure for consistency and transparency in reporting. For further information on Nature Portfolio policies, see our Editorial Policies and the Editorial Policy Checklist.

Statistics

For all statistical analyses, confirm that the following items are present in the figure legend, table legend, main text, or Methods section.

n/a	Confirmed	
☐	☒	The exact sample size (*n*) for each experimental group/condition, given as a discrete number and unit of measurement
☐	☐	A statement on whether measurements were taken from distinct samples or whether the same sample was measured repeatedly
☒	☐	The statistical test(s) used AND whether they are one- or two-sided<br>*Only common tests should be described solely by name; describe more complex techniques in the Methods section.*
☒	☐	A description of all covariates tested
☒	☐	A description of any assumptions or corrections, such as tests of normality and adjustment for multiple comparisons
☐	☒	A full description of the statistical parameters including central tendency (e.g. means) or other basic estimates (e.g. regression coefficient) AND variation (e.g. standard deviation) or associated estimates of uncertainty (e.g. confidence intervals)
☒	☐	For null hypothesis testing, the test statistic (e.g. *F*, *t*, *r*) with confidence intervals, effect sizes, degrees of freedom and *P* value noted<br>*Give P values as exact values whenever suitable.*
☒	☐	For Bayesian analysis, information on the choice of priors and Markov chain Monte Carlo settings
☒	☐	For hierarchical and complex designs, identification of the appropriate level for tests and full reporting of outcomes
☒	☐	Estimates of effect sizes (e.g. Cohen's *d*, Pearson's *r*), indicating how they were calculated

Our web collection on statistics for biologists contains articles on many of the points above.

Software and code

Policy information about availability of computer code

Data collection	We used AutoEMation (version 2.0) to collect cryo-EM datasets of DmDcr-2/R2D2/LoqsPD and 50bp-dsRNA, written by Dr. Jianlin Lei at Tsinghua University. 19bp-dsRNA dataset was collected by EPU (version 2.10) of Thermo Fisher Scientific.
Data analysis	We used MotionCor2 (version 1.1.0) to correct the beam-induced motion of cryo-EM micrographs. The CTF values of these motion-corrected micrographs were determined by CTFFIND4 algorithm (version 4.15). We used Relion (version 3.1.3) and Cryo-SPARC (version 3.20) to perform image analysis and 3D reconstruction. Atomic models were built and refined using Coot (version 0.9.8) and Phenix (version 1.19).The structural analysis was performed in UCSF Chimera (version 1.13.1) and ChimeraX (version 1.4). All these softwares are open-source except Cryo-SPARC, but it is free for educational users.

For manuscripts utilizing custom algorithms or software that are central to the research but not yet described in published literature, software must be made available to editors and reviewers. We strongly encourage code deposition in a community repository (e.g. GitHub). See the Nature Portfolio guidelines for submitting code & software for further information.

Data

Policy information about availability of data

All manuscripts must include a data availability statement. This statement should provide the following information, where applicable:

- Accession codes, unique identifiers, or web links for publicly available datasets
- A description of any restrictions on data availability
- For clinical datasets or third party data, please ensure that the statement adheres to our policy

All the cryo-EM maps and models have been deposited in the wwPDB OneDep System. The EMD accession codes of Dimer state from 50bp-oligomer, Trimer state 19bp-oligomer and Trimer state 50bp-oligomer are 34707, 34708, 34709. The PDB ID codes of Dimer state from 50bp-oligomer, Trimer state 19bp-oligomer are 8HF0 and 8HF1 respectively. Other parameters are listed in Extended Data Table 1. Other structural models cited in this study for analysis (7W0A, 7W0D, 4WYQ and 7V6C) are also accessible through the PDB. All other data or materials can be obtained from the corresponding author upon request.

Research involving human participants, their data, or biological material

Policy information about studies with human participants or human data. See also policy information about sex, gender (identity/presentation), and sexual orientation and race, ethnicity and racism.

Reporting on sex and gender	None
Reporting on race, ethnicity, or other socially relevant groupings	None
Population characteristics	None
Recruitment	None
Ethics oversight	None

Note that full information on the approval of the study protocol must also be provided in the manuscript.

Field-specific reporting

Please select the one below that is the best fit for your research. If you are not sure, read the appropriate sections before making your selection.

☒ Life sciences ☐ Behavioural & social sciences ☐ Ecological, evolutionary & environmental sciences

For a reference copy of the document with all sections, see nature.com/documents/nr-reporting-summary-flat.pdf

Life sciences study design

All studies must disclose on these points even when the disclosure is negative.

Sample size	For cryo-EM data, we did not perform sample size calculations. We pre-collected a portion of the data, such as for one day, and then analyzed it. We ceased further data processing and collection when we believed that the obtained maps could address all the conclusions required in the manuscript. For the in vitro dicing assay, we conducted three replicates and consistently obtained the same results each time.
Data exclusions	For cryo-EM reconstruction, particles grouped in bad classes with poorly defined features were excluded, because these particles were normally denatured or dissociated samples, which were useless or even harmful for high-resolution 3D reconstruction.
Replication	The in vitro dicing assay were repeated three times (Extended Data Fig. 6b).
Randomization	For cryo-EM reconstruction, samples were allocated random, including the particle-motion and structural determination.
Blinding	Blinding was not applicable to this study as visual inspection was necessary to evaluate the data quality of both cryo-EM reconstruction and other experiments.

Reporting for specific materials, systems and methods

We require information from authors about some types of materials, experimental systems and methods used in many studies. Here, indicate whether each material, system or method listed is relevant to your study. If you are not sure if a list item applies to your research, read the appropriate section before selecting a response.

Materials & experimental systems

n/a	Involved in the study
☒	☐ Antibodies
☐	☒ Eukaryotic cell lines
☒	☐ Palaeontology and archaeology
☒	☐ Animals and other organisms
☒	☐ Clinical data
☒	☐ Dual use research of concern
☒	☐ Plants

Methods

n/a	Involved in the study
☒	☐ ChIP-seq
☒	☐ Flow cytometry
☒	☐ MRI-based neuroimaging

Eukaryotic cell lines

Policy information about cell lines and Sex and Gender in Research

Cell line source(s)	SF9 and S2, obtained from Invitrogen.
Authentication	The cell lines were obtained from commercial source and none of lines used were authenticated.
Mycoplasma contamination	Cell lines in this study were negative to mycoplasma by detection by PCR.
Commonly misidentified lines (See ICLAC register)	No commonly misidentified cell lines were used in this study.
